# Surgical Outcomes of Cerebellopontine angle Tumors in 50 Cases

**Published:** 2015-01

**Authors:** Faramarz Memari, Fatemeh Hassannia, Seyed Hamid Reza Abtahi

**Affiliations:** 1 *Department of Otorhinolaryngology Head and Neck Surgery, Hazrate Rasul Medical Center, Iran University of Medical Sciences. Tehran, Iran.*; 2 *Department of Otorhinolaryngology Head and Neck Surgery, Isfahan University of Medical Sciences,Isfahan, Iran*.

**Keywords:** Acoustic neuroma, Cerebellopontine angle tumors, Retrosigmoid approach, Translabyrinthine approach.

## Abstract

**Introduction:**

To report our experience with a large series of surgical procedures for removal of cerebellopontine angle (CPA) tumors using different approaches.

**Materials and Methods:**

This was a retrospective analysis of 50 patients (mean age, 49 years) with CPA tumors (predominantly acoustic neuroma) who underwent surgical removal using appropriate techniques (principally a translabyrinthine approach) during a 4-year period.

**Results:**

One death occurred during this study. There were nine cases (18%) of cerebrospinal fluid leak, and five patients (10%) were diagnosed as having bacterial meningitis. Complete gross tumor removal was not achieved in four patients (8%). Facial nerve function as measured by the House Brackmann system was recorded in all patients 1 year following surgery: 32% had a score of 1 or 2; 26% had a score of 3 or 4; and 8% had a score of 5 or 6. Other complications included four cases of wound infection.

**Conclusion:**

The translabyrinthine approach was predominantly used in our series of CPA tumors, and complication rates were comparable with other large case series.

## Introduction

Acoustic neuromas are the most common tumors of the cerebellopontine angle (CPA), accounting for more than 90% of all such tumors. Other tumors of the CPA include meningioma (3%), primary cholesteatoma, and facial nerve schwannoma. Various techniques are available for the resection of CPA tumors, including translabyrinthine, retrosigmoid, suboccipital, retrolabyrinthine, transcochlear, transotic and middle fossa approaches (1-3).

Acoustic neuromas, also known as vestibular schwannomas, account for approximately 6% of all brain tumors. Acoustic neuromas are located in the cerebellopontine angle and are typically benign fibrous growths that arise from one of the vestibular divisions of the eighth cranial nerve or vestibulocochlear nerve. As the tumors increase in size, they interfere with surrounding structures involved with vital functions such as swallowing, coordinated movement, hearing, balance, facial movements and sensation. In the majority of cases, these tumors grow slowly over a period of years (4-7). 

Several treatment modalities are currently used for the treatment of acoustic neuromas. Until the previous decade, surgical removal of the tumor was the standard form of therapy. Patients now also have the option of undergoing stereotactic radiosurgery or gamma-knife surgery to halt the growth of the tumor. Some patients might also be candidates for a combination of these therapies (8).

Approximately 20–25% of all cases of acoustic neuroma are followed by observation only without any intervention.

Since its introduction in 1960 (1), the translabyrinthine approach has become an increasingly popular method of excising acoustic neuromas. Advantages of this approach include a low complication rate, particularly with regard to facial nerve function (2), and total tumor removal in the vast majority of cases (2,3). Moreover, the technique is safe and effective, even with the largest of tumors (4).

## Materials and Methods

This is a retrospective study of 50 patients with CPA (predominantly acoustic neuroma) referring to the ear, nose, and throat (ENT) clinic of Hazrate Rasul Medical Center during a 4-year period and undergoing surgical removal using appropriate techniques (principally a translabyrinthine approach). Patients were selected for surgery after considering a number of factors including age, tumor size, preoperative hearing (ipsilateral as well as contralateral), patient’s general condition, and patient expectations. The different treatment options (no intervention, radiotherapy,or surgery) were discussed with the patients.

A translabyrinthine approach was used principally for tumors >2 cm in diameter and in patients with unserviceable preoperative hearing. Unserviceable was defined as SRT (Speech Reception Threshold) more than 50 db and SDS (Speech Discrimination Score) less than 50%. A retrosigmoid approach was preferred for patients with good preoperative hearing and tumors <2 cm in diameter. In one patient, a transotic approach was preferred because of an unfavorable venous anatomy. The same surgeon (senior author) performed the procedure on all patients. 

## Results

**Fig 1:**
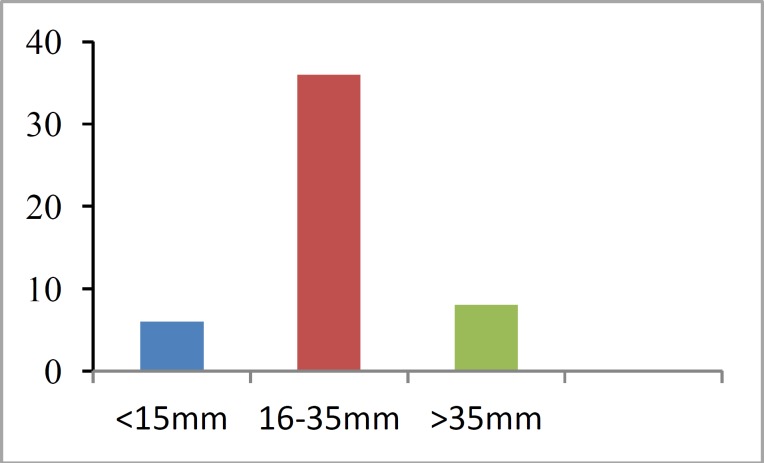
Distribution of tumors by size

A total of 50 patients with a mean age of 49 years (range, 19–87 years) were included in this study. There was a slight male predominance of 55%. The pathology in the majority of cases was acoustic neuroma; in four patients, the pathology was meningi- oma. The mean tumor size was 24 mm, ranging from <15 mm to >35 mm (Fig. 1).

Forty-seven patients (94%) presented with tinnitus, and vertigo was present in 30 patients (60%). The preoperative pure tone average was 60 db. Fourteen patients (28%) had a pure tone average >50 db and a word recognition score >60%. Of these, the majority of patients (11 patients) had tumors >15 mm in maximum diameter. Seven patients had cranial nerve 7 paresis preoperatively(maximum House Brackmann score, 4). Cranial nerve 5 involvement was present in four patients preoperatively, all of whom had tumor size >30 mm. Two patients had cranial nerve 10, 11, and 12 involvement preoperatively and two patients had hemiplasia preoperatively (Table. 1).

**Table 1:** tumor size and preoperative symptoms

**Tumor size**	**Facial nerve paresis**	**Unservisable hearing**	**tinnitus**	**vertigo**	**CN 5 involvement**	**CN 10,11,12**	**hemiplagia**
1-15mm (6)	_	3(50%)	5(83.3%)	2(33.3%)	_	_	_
16-35mm (36)	2(5.5%)	26(72%)	35(97%)	24(66.6%)	1(2.7%)	_	_
>35mm(8)	5(62.5%)	7(87.5%)	7(87.5%)	4(50%)	3(37.5%)	2(25%)	2(25%)

Thirty-four patients underwent translabyrinthine surgery; in 14 patients, a retrosigmoid approach was preferred. In one patient, the tumor was removed via a transotic approach because of an unfavorable venous anatomy. A retrosig- moid and transpetrosal approach was adopted in one patient with a particularly large meningioma (49 mm). All patients had unserviceable hearing postoperatively. Forty-six patients had a histologically proven diagnosis of acoustic neuroma, while in four patients the histologic diagnosis was meningioma.

*Mortality*

There was one death in this series (mortality rate, 2%) which was due to intracranial hemorrhage.

*Neurologic complications (excluding facial nerve injuries)*

In one patient, seizure occurred postoperatively due to increased intracranial pressure. Transient cranial nerve 11 and 12 palsy occurred in one patient, and this was managed via a retrosigmoid approach. Intracranial hemorrhage occurred in one patient, and one patient developed insipidus diabetes postoperatively.

*Facial nerve function*

Facial nerve function was graded according to the House Brackmann scale in the immediate postoperative period and after 1 year. Thirty-two patients (64%) had a Grade 1 or 2 score at 1 year, while 26% had a score of 3 or 4, and 8% had a score of 5 or 6. The results are summarized in (Table. 2). 

**Table 2:** tumor size and final facial nerve outcome by House-Brakmann grade

**Tumor size**	**Grade1**	**Grade2**	**Grade3**	**Grade4**	**Grade5**	**Grade6**	**total**
1-15mm	5(83%)	1(17%)					6
16-35mm	16(45%)	4(11%)	6(17%)	5(14%)	3(8%)	2(5%)	36
>35mm	1(12.5%)	1(12.5%)		2(25%)	2(25%)	2(25%)	8

As expected, there was a significant correlation between tumor size and facial nerve outcome, with larger tumors yielding worse outcomes. Table 2 illustrates this relationship, comparing final facial nerve outcome with tumor size (Pearson r=0.29, P=0.001). Of all patients, 34 were monitored for facial nerve function during surgery and 17 were not.

*Cerebrospinal fluid (CSF) leakage*

CSF leakage occurred in nine patients (18%). The leak presented either as rhinorrhea or at the incision site. These cases were handled in one of three different ways: (1) conservatively with application of a pressure dressing and bed rest; (2) with lumbar drain placement; or (3) with surgical repair. The mean tumor size in patients with CSF leaks was 28 mm, compared with 20 mm in patients without CSF leaks. This difference was statistically significant. We found that tumor size can influence the development of CSF leaks (Table. 3).

**Table 3:** tumor size and postoperative symptoms

**Tumor size**	**CSF leak**	**meningitis**	**Residual tumor**	**Death**	**Decreased LOC**
<15 mm(6)	1(12.5%)	-		_	_
16-35mm(36)	6(16%)	3(8.3%)	3(8.3%)	_	_
>35mm(8)	2(30%)	2(25%)	1(12.5%)	1(12.5%)	2(25%)

Of nine patients with CSF leakage, four were managed via surgical techniques other than a translabyrinthine approach (a transotic approach in one patient and a retrosigmoid approach in three patients). Indeed CSF leakage occurred in only 5 cases (14%) among a total of 34 patients managed via a translabyrinthine approach (Table. 4).

**Table 4:** different approaches and postoperative symptoms

**Surgical approach**	**CSF leak**	**Residual tumor**	**Facial nerve paresis (more than grade 4 HB after one year)**	**Death**
TLA (34)	5(14%)	3(8.8%)	13(38%)	_
Retrosigmoid (14)	3(21%)	1(7%)	3(21%)	_
Transotic (1)	1	_	_	_
Retrosigmoid transpetrosal (1)	_	_	_	1

*Meningitis*

Bacterial meningitis was indicated by the presence of the classic symptoms and was confirmed by spinal fluid analysis. 

Of the five patients (10%) diagnosed as having bacterial meningitis in this series, four had concomitant CSF leakage which presented as rhinorrhea, and these patients were treated with lumbar drainage. All cases of meningitis resolved with intravenous antibiotic therapy without further sequelae. 

*Residual tumor*

Complete gross tumor removal was achieved in all but four patients (92%). Of the patients with complete tumor removal, none had evidence of recurrence on a computed tomography(CT)scan or magnetic resonance imaging (MRI) beyond 1 year.

*Miscellaneous*

Forty-seven patients (94%) had tinnitus preoperatively; which decreased to 21 postoperatively. Thirty patients (60%) suffered from vertigo/disequilibrium that decreased to 11 postoperatively. Wound infection was seen in four patients (8%), of whom three cases were associated with CSF leak and meningitis.

## Discussion

Our findings confirm the generally-held notion that the translabyrinthine approach is a safe and effective method for excising acoustic neuromas. Most importantly, there were no deaths among patients undergoing a translabyrinthine approach. The only death in this series was a 51-year old male who had a large tumor (4.9 cm) and was managed via a retrosigmoid plus transpetrosal approach. This patient developed a decreased level of consciousness due to intracranial hemorrhage postoperatively. Other studies have reported mortality rates of 1–5% (2). Typically, the cause of death is a severe neurovascular insult. In our study, there were two patients with a decreased level of consciousness postoperatively. In one of them, the condition worsened and led to death. In both cases, the tumor was very large (4.9 and 4 cm, respectively). While increased tumor size clearly leads to a higher complication rate in general, it should be noted that only two of our eight patients with large tumors (>35 mm) had a neurovascular complication. This finding is in accordance with a recent study that demonstrated the safety of the translabyrinthine approach for large acoustic neuromas. In that study, tumors >4.0 cm had a 4.8% neurovascular complication rate (4).

Rates of CSF leakage have fallen dramatically according to refinement of surgical techniques. Previously reported rates of 20% have declined with the use of fat packing into the mastoid region and obliteration of the Eustachian tube and middle ear space (6). In our series, CSF leaks developed in 18% of patients; however four of these cases were managed via approaches other than the translabyrinthine technique (transotic and retrosigmoid approaches in one and three cases, respectively). Indeed CSF leakage occurred in only five (14%) cases from a total of 34 patients managed via the translabyrinthine approach (Table. 4). This rate is comparable with other recent series that have placed rates at 6–16% (2,5,6). We found that tumor size can influence the development of CSF leaks. This finding is in contrast with observations noted in other reports (7). Other factors such as delayed wound healing and episodes of increased intracranial pressure may also play a role.

Data regarding facial nerve preservation are presented in Table 2. The majority of patients had normal or near-normal function at 1 year, with 64% exhibiting Grade 1 or 2 facial nerve function. These rates are comparable with those of other reported series (2,9,10). As shown in Table 2, there is a clear correlation between increasing tumor size and worsening facial nerve outcome. This is not surprising, as larger tumors tend to become more intimately involved with the facial nerve or significantly alter its course. Approximately two-thirds of patients with large tumors will experience some permanent facial weakness following surgery (11). The use of facial nerve monitoring has become the standard care for these procedures. 

Bacterial meningitis developed in five cases (10%), four of which developed in the presence of CSF leaks. These data are suggestive of a relationship and support the urgency of rapidly closing a CSF leak. Other authors have found no statistical association between CSF leakage and meningitis (6,9).

## Conclusion

As the translabyrinthine technique is the most familiar approach for acoustic neuroma surgery among otologists, it is important that surgeons become aware of common complications associated with

this technique.
